# The Antithrombotic Agent Pterostilbene Interferes with Integrin α_IIb_β_3_-Mediated Inside-Out and Outside-In Signals in Human Platelets

**DOI:** 10.3390/ijms22073643

**Published:** 2021-03-31

**Authors:** Wei-Chieh Huang, Kao-Chang Lin, Chih-Wei Hsia, Chih-Hsuan Hsia, Ting-Yu Chen, Periyakali Saravana Bhavan, Joen-Rong Sheu, Shaw-Min Hou

**Affiliations:** 1 Graduate Institute of Medical Sciences, College of Medicine, Taipei Medical University, Taipei 110, Taiwan; m120107013@tmu.edu.tw (W.-C.H.); gaujang@mail2000.com.tw (K.-C.L.); d119106003@tmu.edu.tw (C.-W.H.); T014913@ms.skh.org.tw (C.-H.H.); 2 Chi Mei Medical Center, Department of Neurology, Tainan 710, Taiwan; 3 Translational Medicine Center, Shin Kong Wu Ho-Su Memorial Hospital, Taipei 111, Taiwan; 4 Department of Pharmacology, School of Medicine, College of Medicine, Taipei Medical University, Taipei 110, Taiwan; y0513260323@tmu.edu.tw; 5 Department of Zoology, Bharathiar University, Coimbatore, Tamil Nadu 641046, India; bhavan@buc.edu.in; 6 Department of Cardiovascular Center, Cathay General Hospital, Taipei 106, Taiwan; 7 Division of Cardiovascular Surgery, Department of Surgery, School of Medicine, College of Medicine, Fu Jen Catholic University, New Taipei City 242, Taiwan

**Keywords:** arterial thrombosis, hydroxyl radicals, integrin α_IIb_β_3_, platelet aggregation, pterostilbene, resveratrol derivative

## Abstract

Platelets play a crucial role in the physiology of primary hemostasis and pathological processes such as arterial thrombosis; thus, developing a therapeutic target that prevents platelet activation can reduce arterial thrombosis. Pterostilbene (PTE) has remarkable pharmacological activities, including anticancer and neuroprotection. Few studies have reported the effects of pterostilbene on platelet activation. Thus, we examined the inhibitory mechanisms of pterostilbene in human platelets and its role in vascular thrombosis prevention in mice. At low concentrations (2–8 μM), pterostilbene strongly inhibited collagen-induced platelet aggregation. Furthermore, pterostilbene markedly diminished Lyn, Fyn, and Syk phosphorylation and hydroxyl radical formation stimulated by collagen. Moreover, PTE directly hindered integrin α_IIb_β_3_ activation through interfering with PAC-1 binding stimulated by collagen. In addition, pterostilbene affected integrin α_IIb_β_3_-mediated outside-in signaling, such as integrin β_3_, Src, and FAK phosphorylation, and reduced the number of adherent platelets and the single platelet spreading area on immobilized fibrinogen as well as thrombin-stimulated fibrin clot retraction. Furthermore, pterostilbene substantially prolonged the occlusion time of thrombotic platelet plug formation in mice. This study demonstrated that pterostilbene exhibits a strong activity against platelet activation through the inhibition of integrin α_IIb_β_3_-mediated inside-out and outside-in signaling, suggesting that pterostilbene can serve as a therapeutic agent for thromboembolic disorders.

## 1. Introduction

Arterial thrombosis can cause cardiovascular diseases (CVDs), including myocardial infarction, atherosclerosis, ischemic stroke, venous thromboembolism, and peripheral artery diseases, which are the leading causes of mortality worldwide. When vascular subendothelial connective tissues are exposed due to injury, platelet adhesion and aggregation are critical events that aid platelet plug formation and halt bleeding (hemostasis). Although the main role of platelets is to prevent blood loss following tissue injury, platelets are responsible for pathogenic thrombus formation which causes vascular thromboembolic diseases [1].

Collagen contained in the basement membrane induces a change in shape from discoid to spheroid with pseudopodic platelet projections. Classically, platelet activation is induced by collagen or soluble platelet agonists, leading to the activation of platelet adhesion receptors, mainly integrin α_IIb_β_3_, which mediates platelet adhesion and aggregation [2]. In resting platelets, integrin α_IIb_β_3_ exists in a low activation state and is unable to interact with its specific ligands such as fibrinogen and fibronectin. Platelet activation stimulated by agonists induces a conformational change in integrin α_IIb_β_3_, enabling it to bind to its ligands, thus resulting in the onset of platelet aggregation; this process is recognized as integrin α_IIb_β_3_ inside-out signaling [2]. Moreover, the binding of fibrinogen to directly activate integrin α_IIb_β_3_ initiates a series of intracellular signaling events, such as the tyrosine phosphorylation of proteins and the reorganization of the cytoskeleton, which are referred to as outside-in signaling [2]. These outside-in reactions, originating in integrin α_IIb_β_3_ bound to fibrinogen, are required for maximal secretion, procoagulation, and clot retraction [2].

**Figure 1:**
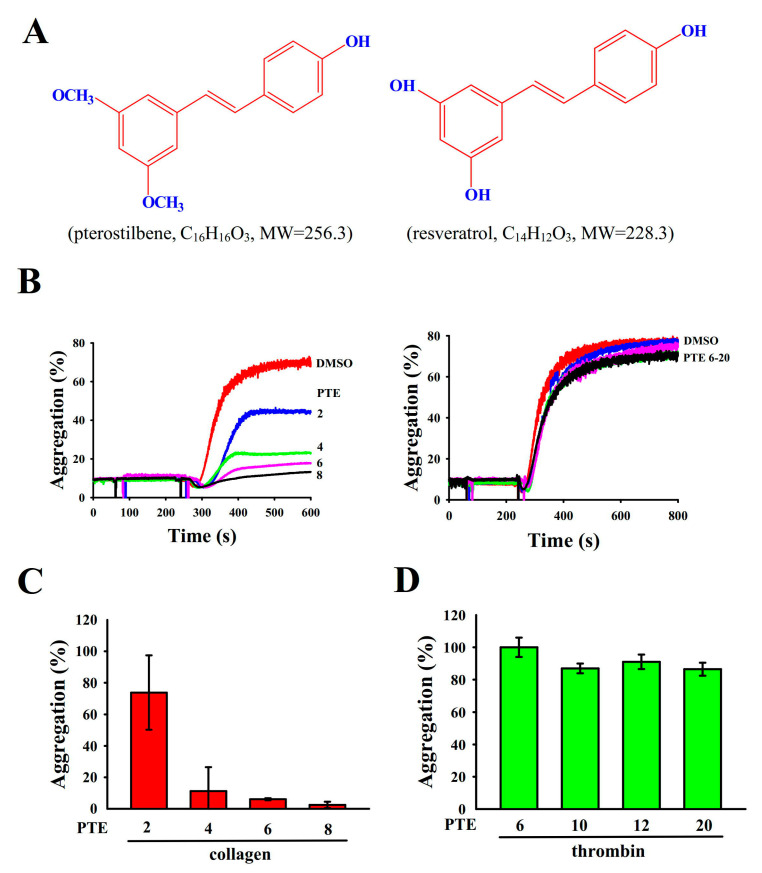
Inhibitory profiles of the effects of pterostilbene (PTE) on platelet aggregation stimulation by collagen and thrombin. (**A**) Chemical structures of PTE (C_16_H_16_O_3_) and resveratrol (C_14_H_12_O_3_). (**B**) Washed human platelets (3.6 × 10^8^ cells/mL) were preincubated with a solvent control (0.1% DMSO) or PTE (2–20 μM) and subsequently treated with either collagen (1 μg/mL) or thrombin (0.01 U/mL) to stimulate platelet aggregation. (**C**,**D**) Concentration–response histograms of PTE demonstrating its inhibitory activity for platelet aggregation stimulated by collagen (%). Data are presented as the mean ± standard deviation (*n* = 4).

Pterostilbene (PTE; trans-3,5-dimethoxy-4′-hydroxystilbene; Figure 1A), a natural stilbenoid occurring in grapes and berries, is a dimethylated analog of resveratrol [3]. PTE exhibits several remarkable pharmacological activities [4] including antiaging, anticancer, anti-diabetes, and neuroprotection. In addition, Park et al. [5] demonstrated that PTE inhibited the PDGF-BB-induced cell growth of vascular smooth muscle cells in rats through inhibition of the Akt-dependent pathway. Furthermore, PTE prevented atherosclerosis through regulation of the Nrf2-mediated TLR-4/MyD88/NF-κB pathway in rats [6]. For antiplatelet activity, Messina et al. [7] reported that PTE markedly diminishes collagen-stimulated platelet aggregation. Moreover, we observed that resveratrol exhibits potent antiplatelet activity through the inhibition of the p38 MAPK-phospholipase A_2_ cascade, as described previously [8]. Our initial screening exhibited that PTE (1–6 μM) is highly effective in inhibiting collagen-stimulated platelet aggregation in humans. However, few studies have reported the effects of PTE on platelet activation. Therefore, in this study, we elucidated PTE mechanisms underlying platelet activation both ex vivo and in vivo to support the scientific rationale for its clinical use.

## 2. Results

### 2.1. Inhibitory Profiles of PTE in Platelet Aggregation Stimulated with Collagen and Thrombin in Humans

PTE (2–8 μM; molecular weight: 256.3; C_16_H_16_O_3_) concentration dependently diminished human platelet aggregation stimulated by collagen (1 μg/mL) but not by thrombin (0.01 U/mL), even at concentrations up to 20 μM (Figure 1B–D). Therefore, PTE showed more powerful activity against collagen stimulation than thrombin. Moreover, aspirin (20, 50, and 100 μM) concentration dependently inhibited collagen-stimulated platelet aggregation, with a half maximal inhibitory concentration (IC_50_) of approximately 55 μM (data not shown). Therefore, PTE (IC_50_: 3.5 μM) was approximately 16-fold more potent than aspirin in inhibiting collagen-stimulated platelet aggregation. The solvent control (0.1% DMSO) did not exert any significant effects on platelet aggregation.

### 2.2. Effects of PTE on Glycoprotein VI-Mediated Lyn, Fyn, and Syk Phosphorylation

**Figure 2:**
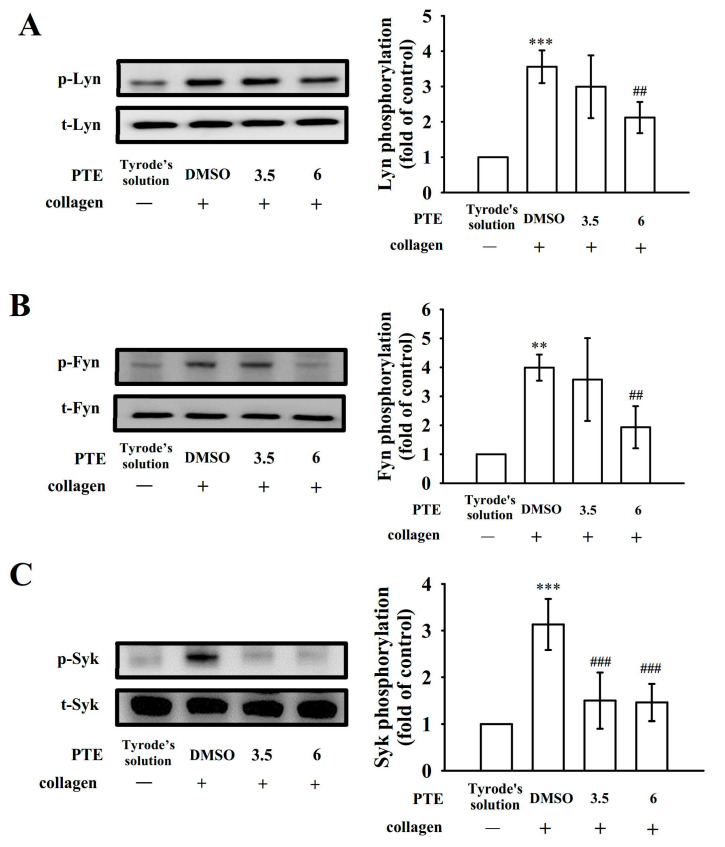
Effectiveness of pterostilbene (PTE) in Lyn, Fyn, and Syk activation in human platelets. Washed platelets (1.2 × 10^9^ cells/mL) were preincubated with 0.1% DMSO or PTE (3.5 and 6 µM), followed by the addition of collagen (1 μg/mL) to trigger (**A**) Lyn, (**B**) Fyn, and (**C**) SyK phosphorylation. Platelets were collected, and subcellular extracts were examined to determine the levels of protein phosphorylation. Data are presented as the mean ± standard deviation (*n* = 4). ** *p* < 0.01 and *** *p* < 0.001, compared with the resting control; ^##^ *p* < 0.01 and ^###^ *p* < 0.001, compared with the 0.1% DMSO-treated group.

The collagen glycoprotein (GP) VI receptor initiates intracellular signaling from the intracellular SH3 binding region, which recruits the active form of Src-family kinases (SFKs) Fyn and Lyn before adhesion to collagen [9], resulting in the activation of the cytosolic tyrosine kinase Syk. The collagen-induced phosphorylation of Lyn, Fyn, and Syk was concentration-dependently inhibited by PTE (3.5 and 6 μM; Figure 2A–C). The corresponding statistical data are presented in the right-hand panels of Figure 2.

### 2.3. Effect of PTE on Integrin α_IIb_β_3_ Activation

**Figure 3:**
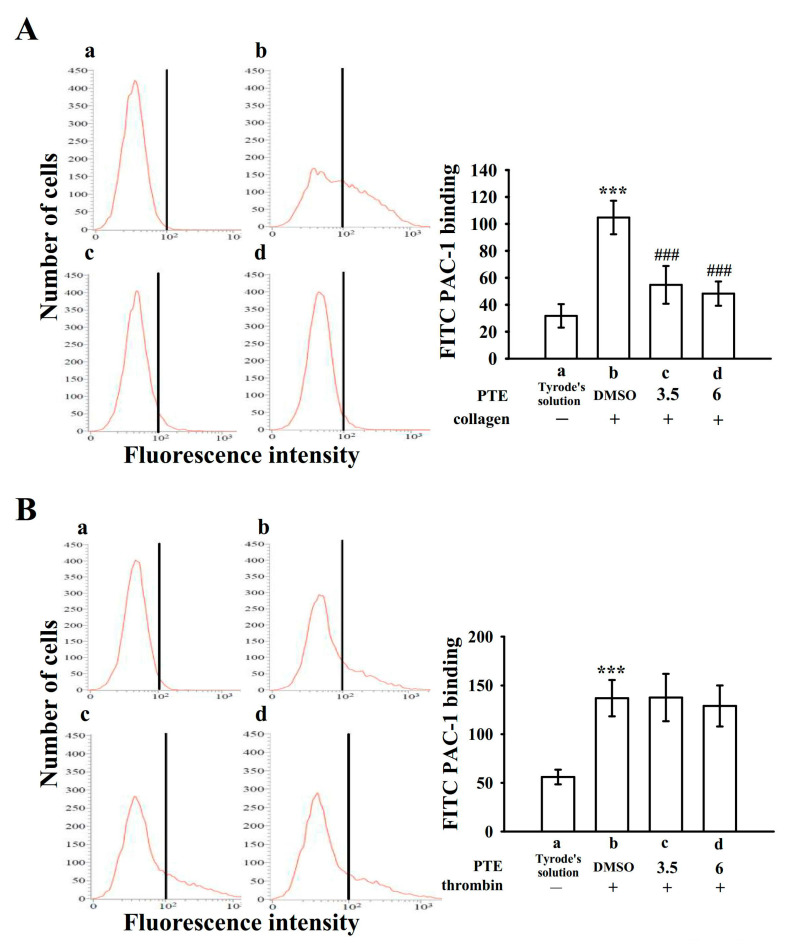
Inhibitory property of pterostilbene (PTE) in integrin α_IIb_β_3_ activation stimulated by either collagen or thrombin. For the flow cytometry analysis, resting platelets (**a**) or platelets were preincubated with the solvent control (**b**, 0.1% DMSO) or PTE (**c**, 3.5 µM; **d**, 6 µM), and fluorescein isothiocyanate-conjugated anti-PAC-1 mAb (2 µg/mL) was added before the addition of (**A**) collagen (1 µg/mL) or (**B**) thrombin (0.01 U/mL). Data are presented as the mean ± standard deviation (*n* = 4). *** *p* < 0.001, compared with the resting control; ^###^ *p* < 0.001, compared with the 0.1% DMSO-treated group.

Platelet aggregation is dependent on the fibrinogen–integrin α_IIb_β_3_ interaction; nevertheless, integrin α_IIb_β_3_ inactivation can lead to the disaggregation of aggregated platelets [10]. To further define whether PTE can affect integrin α_IIb_β_3_ activation, the binding of the fluorescein isothiocyanate (FITC)-PAC-1 mAb specific for neoepitopes exposed to the activated form of integrin α_IIb_β_3_ was analyzed through flow cytometry. PTE reduced FITC-PAC-1 binding stimulated by collagen but not by thrombin (Figure 3). The corresponding statistical data are presented in the right-hand panels of Figure 3A (resting control, 31.7 ± 8.7; collagen-activated platelets, 104.8 ± 12.5; 3.5 μM PTE, 54.7 ± 14.0; 6 μM PTE, 48.3 ± 9.0; *n* = 4) and Figure 3B (resting control, 56.0 ± 7.5; thrombin-activated platelets, 137.1 ± 18.7; 3.5 μM PTE, 137.6 ± 24.3; 6 μM PTE, 129.2 ± 21.1; *n* = 4).

### 2.4. PTE Restricts Integrin α_IIb_β_3_-Mediated Outside-In Signaling of Protein Kinase Activation

**Figure 4:**
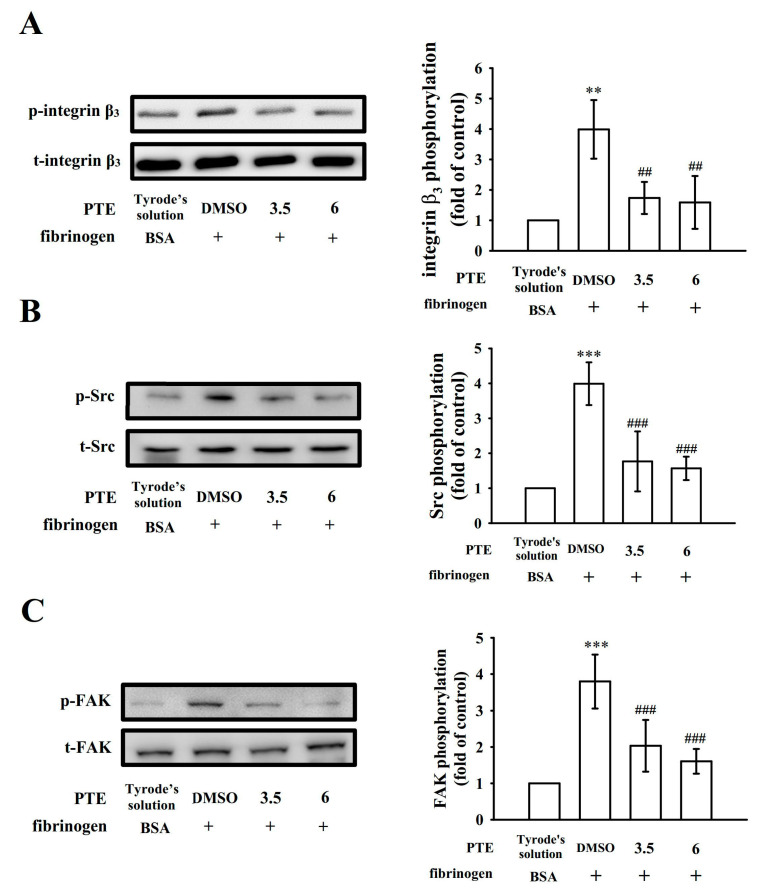
Effects of pterostilbene (PTE) on integrin β_3_, Src, and FAK phosphorylation in platelets exposed to immobilized fibrinogen. Washed platelets were preincubated with the solvent control (0.1% DMSO) or PTE (3.5 and 6 µM) and subsequently activated with immobilized fibrinogen (100 μg/mL) for determining the levels of (**A**) integrin β_3_, (**B**) Src, and (**C**) FAK phosphorylation, as described in the Materials and Methods section. Data are presented as the mean ± standard deviation (*n* = 4). ** *p* < 0.01 and *** *p* < 0.001, compared with the bovine serum albumin (BSA; control); ^##^ *p* < 0.01 and ^###^ *p* < 0.001, compared with the 0.1% DMSO-treated group.

To further elucidate mechanisms through which PTE diminishes integrin α_IIb_β_3_-mediated outside-in signaling, integrin β_3_ phosphorylation, a vital indicator of outside-in signaling, was studied. We examined integrin β_3_ phosphorylation in platelets exposed to immobilized fibrinogen and observed that integrin β_3_ phosphorylation was attenuated in the presence of PTE (3.5 and 6 μM; Figure 4A). Moreover, PTE significantly reduced the immobilized fibrinogen-induced phosphorylation of Src and FAK (Figure 4B,C). The corresponding statistical data are presented in the right-hand panels of Figure 4. These data suggest that PTE affected integrin α_IIb_β_3_-mediated outside-in protein kinase phosphorylation in human platelets.

### 2.5. PTE Limits Integrin α_IIb_β_3_-Mediated Outside-In Signaling of Platelet Adhesion and Spreading As Well As Fibrin Clot Retraction

**Figure 5:**
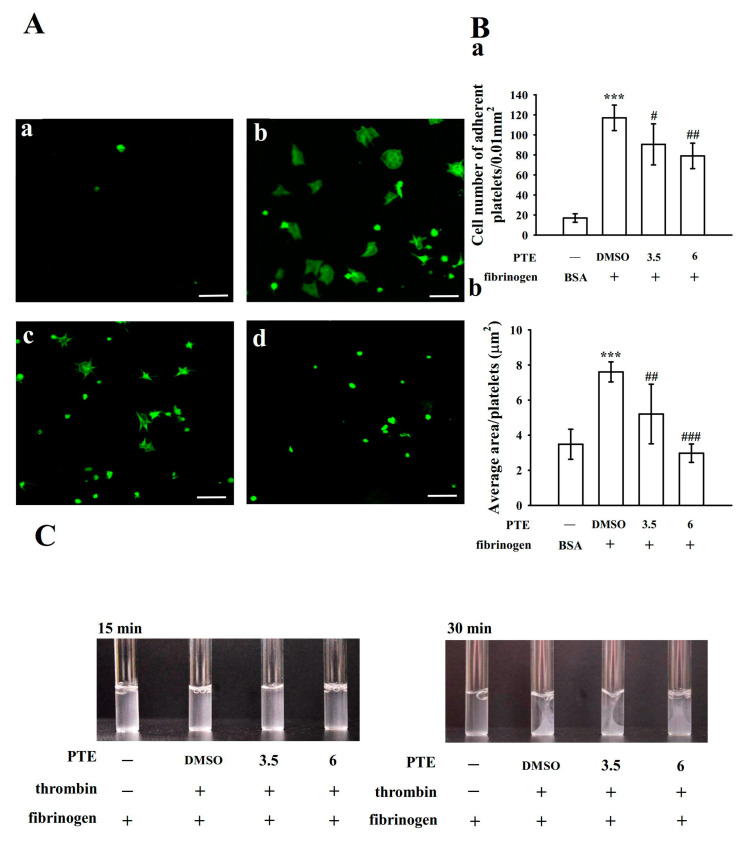
Inhibitory activity of pterostilbene (PTE) on platelet adhesion and spreading on immobilized fibrinogen as well as fibrin clot retraction. (**A**) Washed platelets were allowed to spread on the (**a**) BSA- or (b–d) fibrinogen-coated surfaces in the presence of the (**b**) solvent control (0.1% DMSO) or PTE (**c**, 3.5 μM; **d**, 6 μM) and subsequently labeled with fluorescein isothiocyanate–phalloidin, as described in the Materials and Methods section. Scale bar, 10 µm. Plot of (**B**) the number of adherent platelets per 0.01 mm^2^ (**a**), and the average spreading surface area of individual platelets in six sight views (**b**). (**C**) Washed platelets (3.6 × 10^8^ cells/mL) were suspended in Tyrode’s solution containing 2 mg/mL fibrinogen and 1 mM CaCl_2_ with the solvent control (0.1% DMSO) or PTE (3.5 and 6 μM). Clot retraction was initiated with thrombin (0.01 U/mL) at 37 °C. Images were photographed at 15 and 30 min intervals by using a digital camera. Profiles in (**C**) are representative of four similar experiments. Data are presented as the mean ± standard deviation (*n* = 4). *** *p* < 0.001, compared with the BSA (control); ^#^ *p* < 0.05, ^##^ *p* < 0.01 and ^###^ *p* < 0.001, compared with the 0.1% DMSO-treated group.

Platelet staining with FITC–phalloidin revealed that significantly more platelets adhered to immobilized fibrinogen than to immobilized BSA (Figure 5(Aa,b)). Marked differences were observed in platelet adhesion and spreading on immobilized fibrinogen for PTE-treated (3.5 and 6 μM) platelets compared with 0.1% DMSO-treated platelets (Figure 5(Ab–d)). As shown in the corresponding statistical data presented in the right-hand panels of Figure 5(Ba), control platelets were predominantly fixed to immobilized fibrinogen compared with immobilized BSA (BSA, 17.0 ± 4.2 platelets/0.01 mm^2^ and fibrinogen, 117.0 ± 12.7 platelets/0.01 mm^2^; *n* = 4); however, PTE (3.5 and 6 μM) treatment concentration dependently reduced platelet adhesion to the fibrinogen-coated surface (3.5 μM, 90.5 ± 20.5 platelets/0.01 mm^2^ and 6 μM, 79.0 ± 12.7 platelets/0.01 mm^2^; *n* = 4). In addition, compared with 0.1% DMSO-treated platelets (7.6 ± 0.6 μm^2^; *n* = 4), the surface coverage of a single platelet treated with PTE was reduced significantly (3.5 μM, 5.2 ± 1.7 μm^2^ and 6 μM, 3.0 ± 0.5 μm^2^; *n* = 4; Figure 5(Bb)).

Fibrin clot retraction by fibrin polymers, the final step in thrombus formation, is essential for aggregate stabilization and a paradigm of integrin α_IIb_β_3_ outside-in signaling [11]. The clot was retracted by adding thrombin into a solution containing fibrinogen in the presence of PTE- or 0.1% DMSO-treated human platelets as demonstrated in Figure 5C. Clot retraction was more apparent after 30 min incubation than after 15 min incubation in 0.1% DMSO-treated platelets, whereas it was substantially reduced in PTE-treated (3.5 and 6 μM) platelets. This finding indicated that PTE induced a deficit in the ability of platelets to mediate stable interactions with a fibrin matrix, reducing fibrin clot retraction. Overall, these data suggest that PTE affects integrin α_IIb_β_3_-mediated outside-in signaling.

### 2.6. Regulatory Activities of PTE in Hydroxyl Radical Formation

**Figure 6:**
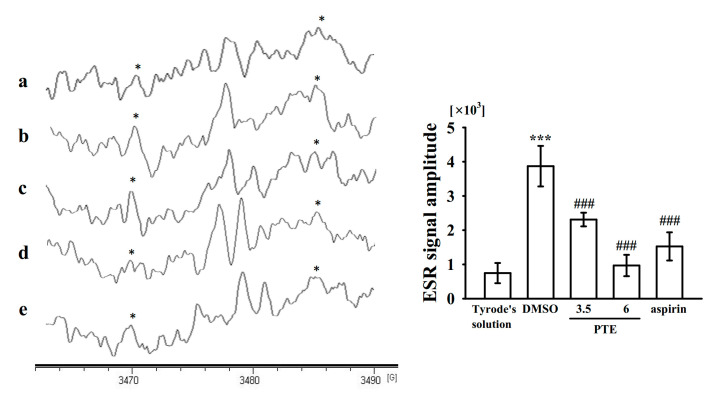
Activity of pterostilbene (PTE) on hydroxyl radical formation in human platelets. Washed platelets were incubated with (**a**) Tyrode’s solution only (resting group) or preincubated with (**b**) 0.1% DMSO, PTE (**c**, 3.5 μM; **d**, 6 μM), (**e**) aspirin (100 μM) followed by the addition of collagen (1 μg/mL) to trigger hydroxyl radical (*) formation. Data are presented as the mean ± standard deviation (*n* = 4). *** *p* < 0.001, compared with the Tyrode’s solution only (resting group); ^###^ *p* < 0.001, compared with the 0.1% DMSO-treated group.

Reactive oxygen species (ROS; such as hydrogen peroxide and hydroxyl radicals) derived from platelet activation might amplify platelet reactivity during thrombus formation. However, the regulatory pathways of ROS, especially for hydroxyl radicals during platelet activation, remain obscure. A typical electron spin resonance (ESR) signal of hydroxyl radical formation was triggered by collagen (1 μg/mL) compared with resting platelets (Figure 6a,b; resting, 747 ± 295; collagen-activated, 3870 ± 592). PTE (3.5 and 6 μM) markedly reduced collagen-induced hydroxyl radical formation (Figure 6c,d; 3.5 μM, 2311 ± 200 and 6 μM, 969 ± 321). However, aspirin (100 μM) showed weaker activity in inhibiting this reaction than PTE (Figure 6e, 1526 ± 412; *n* = 4).

### 2.7. Effect of PTE in Vascular Thrombus Formation In Vivo

**Figure 7:**
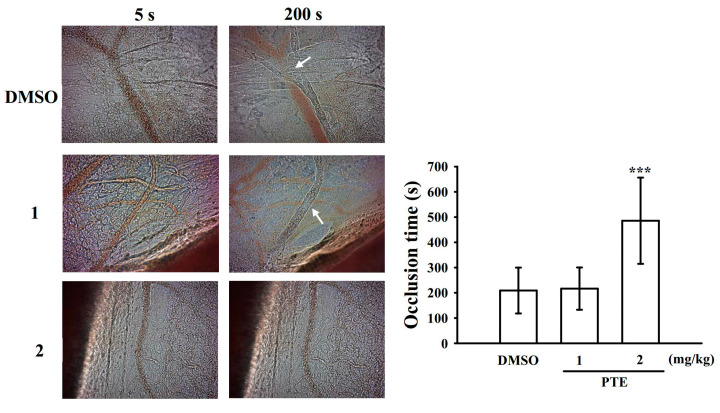
Effect of pterostilbene (PTE) on vascular thrombosis in the mesenteric venules of mice. Mice were administered an intravenous bolus of the solvent control (0.1% DMSO) or PTE (1 and 2 mg/kg), and the mesenteric venules were irradiated to induce microthrombus formation (occlusion time). Microscopic images (400× magnification) of 0.1% DMSO-treated controls and 1 and 2 mg/kg PTE-treated groups were recorded at 5 and 200 s after irradiation, respectively. The photographs shown are representative of eight similar experiments, and white arrows indicate platelet plug formation. Data are presented as the mean ± standard deviation (*n* = 8). *** *p* < 0.001, compared with the 0.1% DMSO-treated group.

The antithrombotic effect of PTE was observed in experimental mice. The occlusion time in the mesenteric microvessels of mice pretreated with fluorescein sodium (15 µg/kg) was approximately 200 s. We administered PTE at 1 or 2 mg/kg after pretreatment with fluorescein sodium; the resulting occlusion times were significantly prolonged after 2 mg/kg PTE treatment compared with those after 0.1% DMSO treatment (control, 209.1 ± 90.9 s vs. 1 mg/kg PTE, 216.6 ± 83.7 s; 2 mg/kg PTE, 485.6 ± 170.8 s; *n* = 8, Figure 7). After irradiation, a thrombotic platelet plug was observed in the mesenteric microvessels at 5 and 200 s in either the 0.1% DMSO- or PTE (1 mg/kg)-treated group (Figure 7; arrows). On PTE (2 mg/kg) administration, platelet plug formation was not observed at 5 and 200 s after irradiation (Figure 7).

## 3. Discussion

The results of this study showed that PTE resulted in high antiplatelet activity in humans. Plant-based polyphenols cause vasoprotection, antiangiogenesis, and antithrombosis in patients with CVDs [12]. Resveratrol, a polyphenol derivative, exhibits valuable activity in controlling heart diseases [13]. However, low oral bioavailability and rapid first-pass metabolism of resveratrol markedly affect its clinical application [14]. In fact, the properties of poor bioavailability and rapid metabolism are common among polyphenols. By contrast, methylated polyphenols exhibit substantially higher intestinal absorption and enhanced hepatic stability [15]. Thus, structural modifications of resveratrol that increase its bioavailability while preserving its beneficial activities are warranted. Structurally, PTE, a naturally occurring dimethyl ether analog of resveratrol, possesses better metabolic stability than resveratrol because it has only one hydroxyl group, whereas resveratrol has three hydroxyl groups (Figure 1A). The dimethyl ether structure of PTE was suggested to increase membrane permeability and enhance its lipophilicity, resulting in better pharmacokinetic profiles than those of resveratrol [16]. Therefore, the bioavailability and plasma levels of PTE were considerably higher than those of the equimolar doses of resveratrol, regardless of the dose or route of administration. The pharmacokinetics of PTE following the daily oral dosing of 56 mg/kg for 14 days in rats found that the blood concentration (Cmax) was approximated at 2550 ng/mL (~10 μM) [17]. The result indicated that the concentration of 3.5 and 6 μM used in this antiplatelet study was reasonable, and can be reached in the circulation after dietary intake. Although normal PTE obtained from natural sources would be insufficient to achieve the required plasma concentration that can inhibit in vivo platelet activation, the long-term intake of sufficient natural food products or nutritional supplements is ideal for preventing atherothrombotic events; thus, PTE may serve as an innovative antithrombotic agent in humans because it exhibits high anti-platelet activity.

Platelets are activated by a variety of physiological stimuli (e.g., thrombin, collagen). In general, these agonists act through specific receptors or act by altering/instigating particular signal transduction pathways associated with other receptors. Thrombin is one of the most potent activators of platelets, and its role in promoting thrombus formation has been clearly established. Thrombin activates platelets through multiple cell-surface receptors, including the GP Ib/V/IX complex and the protease-activated receptors (PARs). Of the four known PAR isoforms, PAR1 and PAR4, are essential for thrombin-induced human platelet activation [18]. Thrombin activates human platelets by cleaving and activating PAR1 and PAR4. In turn, these receptors activate Gq, G_12/13_, and possibly the Gi family, which leads to the activation of phospholipase C, phosphoinositide 3-kinase, and the monomeric G proteins (i.e., Rho); the activation also causes an increase in cytosolic Ca^2+^ concentration and inhibits cyclic AMP formation [18,19]. In addition, platelet adhesion is related to collagen. Platelets can adhere to multiple surfaces including cells and other adhesive proteins; however, initial adhesion is typically to the collagen surface. Collagen is found in the subendothelial space and within the tunica media (middle layer of blood vessels) and tunica adventitia (outermost layer of blood vessels). Therefore, collagen is the most important protein that can interact with platelets and induce activation responses. Apparently, all collagen receptors converge to the platelet tyrosine kinase signaling cascade, which promotes a transient increase in intracellular calcium, platelet aggregation (through integrin α_IIb_β_3_), and granule secretion [20]. Among platelet receptors known to directly interact with collagen, integrin α_2_β_1_ (GP Ia/IIa) and GP VI appear to play a key role and have recently gained the attention of researchers [21]. GP VI is widely recognized as a requisite factor for platelet aggregate formation on a collagen surface under blood flow; integrin α_2_β_1_ is another collagen receptor on endothelial cells and platelets. GP VI belongs to a membrane of the immunoglobulin superfamily, which forms a complex with the Fc receptor γ-chain containing immunoreceptor tyrosine-based activation motifs and is phosphorylated by SFKs such as Fyn and Lyn [22]. In turn, different pathways of protein phosphorylation regulate integrin α_IIb_β_3_ activation through inside-out mechanisms. In the current study, PTE selectively inhibited platelet aggregation induced by collagen rather than that by thrombin, indicating that antiplatelet effects of PTE may interfered with the signal transduction pathway stimulated by collagen, but not by thrombin; however, more experiments are needed to verify the detailed mechanisms of PTE.

The fibrinogen–integrin α_IIb_β_3_ binding belongs to a major component of activated platelets. Integrin α_IIb_β_3_ undergoes conformational changes on activation, generating a unique and specific ligand-binding site for fibrinogen, von Willebrand factor, and fibronectin [2]. PAC-1 reacts with the activation-induced conformational epitope of integrin α_IIb_β_3_ [23], and PAC-1 binding was markedly reversed by PTE treatment stimulated by collagen. In addition, platelets adhered to immobilized fibrinogen and platelet-mediated fibrin clot retraction are involved in integrin α_IIb_β_3_ outside-in signaling [2]. Integrin α_IIb_β_3_-mediated signaling begins immediately after a fibrinogen molecule binds to the integrin α_IIb_β_3_; this outside-in signaling results in the tyrosine phosphorylation of numerous proteins, such as SFK, FAK, and the cytoplasmic tail of integrin β_3_ at Tyr^759^, a process dependent on outside-in signaling and cytoskeleton reorganization [2]. The critical role of integrin β_3_ at Tyr^759^ in platelets was demonstrated in vivo, and its mutation led to bleeding disorder and strongly affected clot retraction responses in vitro [24]. FAK, a cytoplasmic tyrosine kinase located at focal adhesion points, plays a vital role in cytoskeleton regulation and integrin α_IIb_β_3_ activity [25]. Adhesion of platelets to immobilized fibrinogen requires FAK activation through integrin α_IIb_β_3_, and, in turn, FAK activation requires autophosphorylation [25]. In the current study, PTE noticeably abolished platelet adhesion and spreading and clot retraction as well as the phosphorylation of integrin β_3_, Src, and FAK on immobilized fibrinogen in the absence of platelet agonists. Taken together, PTE potentially acts on integrin α_IIb_β_3_ and blocks both integrin α_IIb_β_3_-mediated inside-out and outside-in signaling. By contrast, we do not rule out the possibility that other, as yet unidentified mechanisms could be involved in the PTE-mediated inhibition of platelet activation.

Reactive oxygen species derived from platelet activation, such as hydrogen peroxide and hydroxyl radicals, play an important role in regulating platelet responses in collagen-mediated thrombus formation [26]. Some of the hydrogen peroxide produced in platelets is converted into hydroxyl radicals, which acts as secondary signals that increase [Ca^2+^]i levels during the initial phase of platelet activation [26]. Begonja et al. [27] reported that ROS produced in platelets significantly affected integrin α_IIb_β_3_ activation. The results of our ESR analysis provide direct evidence that PTE scavenges hydroxyl radicals in human platelets. Thus, the PTE-mediated inhibition of thrombogenesis in vivo may involve scavenging free radical formation. After vascular endothelial cell injury, exposure to subendothelial collagen majorly triggers platelet adhesion and aggregation at the injury site, followed by vascular thrombosis. Animal models of vascular thrombosis are necessary to understand the effectiveness of test compounds in disease treatment. An ideal mouse model should technically be simple, quick in operation, and easily reproducible. In a vascular thrombotic mouse model [28], mesenteric venules were continuously irradiated with fluorescein sodium throughout the experimental period, which severely damaged the endothelium, whereas treatment with 2 mg/kg PTE significantly extended the occlusion time. These data are consistent with the fact that platelet aggregation is a crucial factor causing vascular thrombosis. Therefore, PTE can be a potential natural compound for treating thromboembolic disorders.

## 4. Materials and Methods

### 4.1. Materials

PTE (>98%), collagen (type I), fibrinogen, heparin, fluorescein isothiocyanate (FITC)–phalloidin, 5,5-dimethyl-1 pyrroline N-oxide (DMPO), bovine serum albumin (BSA), aspirin, and thrombin were purchased from Sigma (St. Louis, MO, USA). An anti-integrin β_3_ monoclonal antibody (mAb) and anti-phospho-integrin β_3_ (Tyr^759^) polyclonal antibody (pAb) were purchased from Santa Cruz Biotechnology (Santa Cruz, CA, USA). Anti-phospho-Src family (Tyr^416^), anti-phospho-Syk (Tyr^525/526^), and anti-phospho-FAK (Tyr^397^) mAbs and anti-Syk, anti-Src family, and anti-FAK pAbs were purchased from Cell Signaling (Beverly, MA, USA). The FITC-anti-human CD41/CD61 (PAC-1) mAb was obtained from BioLegend (San Diego, CA, USA). Anti-phospho-Fyn (Tyr^530^) pAb, anti-phospho-Lyn (Tyr^497^), anti-Fyn, and anti-Lyn mAbs were obtained from Abcam (Cambridge, UK). A Hybond-P polyvinylidene difluoride membrane, an enhanced chemiluminescence Western blotting detection reagent, horseradish peroxidase-conjugated donkey anti-rabbit immunoglobulin G (IgG), and sheep anti-mouse IgG were purchased from Amersham (Buckinghamshire, UK). PTE suspension was prepared in 0.1% dimethyl sulfoxide (DMSO) and stored at 4 °C.

### 4.2. Platelet Preparation and Aggregation Study

This study complied with the directives of the Helsinki Declaration and was approved by the Institutional Review Board of Taipei Medical University (N201812024). Informed consent was obtained from all human volunteers who participated in this study. Human platelets were washed as described previously [29]. Blood was mixed with acid/citrate/glucose (9:1, *v*/*v*). After centrifugation at 120× *g* for 10 min, the supernatant (platelet-rich plasma) was supplemented with EDTA (2 mM) and heparin (6.4 U/mL), incubated for 5 min at 37 °C, and centrifuged at 500× *g* for 10 min. The platelet pellet was suspended in 5 mL of Tyrode’s solution, pH 7.3 (containing NaCl (11.9 mM), KCl (2.7 mM), MgCl_2_ (2.1 mM), NaH_2_PO_4_ (0.4 mM), NaHCO_3_ (11.9 mM) and glucose (11.1 mM)) and the mixture was incubated for 10 min at 37 °C. After centrifugation of the suspension at 500× *g* for 10 min, the washing procedure was repeated. The washed platelets were finally suspended in Tyrode’s solution containing BSA (3.5 mg/mL). The platelet count was monitored by a Coulter counter (Beckman Coulter, Miami, FL, USA). The final concentration of Ca^2+^ in the Tyrode’s solution was 1 mM. Washed human platelets (3.6 × 10^8^ cells/mL) were incubated with PTE (2–20 μM) or solvent control (0.1% DMSO) for 3 min before stimulation with thrombin (0.01 U/mL) or collagen (1 μg/mL).

### 4.3. Study of Binding Activated Integrin α_IIb_β_3_

Briefly, washed platelets were preincubated with PTE (3.5 and 6 µM) and FITC-conjugated PAC-1 mAb (2 µg/mL) for 3 min and then stimulated with collagen (1 µg/mL). The suspensions were then assayed for fluorescein-labeled platelets on a flow cytometer (FAC Scan system, Becton Dickinson, San Jose, CA, USA). Data were collected from 50,000 platelets per experimental group, and the platelets were identified based on their characteristic forward and orthogonal light-scattering profiles. All experiments were repeated at least four times to ensure reproducibility.

### 4.4. Immunoblotting

Washed platelets (1.2 × 10^9^ cells/mL) were preincubated with PTE (3.5 and 6 µM) or 0.1% DMSO for 3 min, and collagen was subsequently added to trigger activation. The platelet suspensions were lysed and separated through 12% sodium dodecyl sulfate polyacrylamide gel electrophoresis. For another study, dishes (6 cm in diameter) were precoated with fibrinogen (100 µg/mL), kept overnight, and then blocked with 1% BSA. Washed platelets (3.6 × 10^8^ cells/mL) were preincubated with PTE (3.5 and 6 µM) or the solvent control (0.1% DMSO) for 3 min and then poured into immobilized fibrinogen dishes for 60 min. The reaction was then stopped, and the platelets were immediately resuspended in 200 μL of lysis buffer. Several proteins were detected using specific primary antibodies. Respective quantitative results were obtained through quantifying the optical density of protein bands by using a video densitometer and Bio-profil BioLight software, version V2000.01 (VilberLourmat, Marne-la-Vallée, France), and relative protein expression was calculated after normalization to the total protein of interest.

### 4.5. Confocal Microscopy Analysis of Platelet Adhesion and Spreading

Eight-chamber glass tissue-culture slides were coated with either BSA (100 μg/mL) or fibrinogen (100 μg/mL) and left overnight. After washing with phosphate-buffered saline (PBS) twice, the slides were blocked with 1% BSA in PBS for 1 h and then washed again with PBS. Washed platelets (3.0 × 10^8^ cells/mL) preincubated with PTE (3.5 and 6 μM) or the solvent control (0.1% DMSO) were spread on protein-coated surfaces for 45 min. After unbound platelet removal and two washes with PBS, the bound cells were fixed (4% paraformaldehyde), permeabilized (0.1% triton), and stained with FITC-phalloidin (10 μM). All confocal studies were performed using a Leica TCS SP5 microscope equipped with a 63×, 1.40 NA oil immersion objective (Leica, Wetzlar, Germany). The number of platelet adhesion events and the platelet spreading surface area were determined using the NIH ImageJ software (NIH, Bethesda, MD, USA).

### 4.6. Platelet-Mediated Fibrin Clot Retraction

Washed platelets (3.6 × 10^8^ cells/mL) were resuspended in Tyrode’s solution containing 2 mg/mL of fibrinogen and 1 mM CaCl_2_ and then dispensed in 500 μL aliquots in glass tubes designed for aggregation [30]. PTE (3.5 and 6 μM) or the solvent control (0.1% DMSO) was included in the platelet suspension buffer before thrombin (0.01 U/mL)-induced clot retraction without stirring. The reaction was photographed at 15 and 30 min.

### 4.7. Measurement of Hydroxyl Radicals Through Electron Spin Resonance Spectrometry

The electron spin resonance (ESR) method was used to measure hydroxyl radicals by using a Bruker EMX ESR spectrometer, as described previously [30]. In brief, platelet suspensions (3.6 × 10^8^ cells/mL) were preincubated with PTE (3.5 and 6 μM) for 3 min before adding collagen (1 μg/mL). The reaction was allowed to proceed for 5 min before adding DMPO (100 μM). The ESR spectrometer was operated at a power of 20 mW and 9.78 GHz, and a scan range of 100 G and a receiver gain of 5 ×10^4^ were applied [31]. The ESR signal amplitude was quantified using the WIN-EPR, version 921201 supplied by BRUKER-FRANZEN Analytik GmbH (Bremen, Germany).

### 4.8. Measurement of Vascular Thrombus Formation in Mouse Mesenteric Microvessels Irradiated with Sodium Fluorescein

The method applied to a thrombogenic animal model in this experiment conformed to the Guide for the Care and Use of Laboratory Animals (8th edition, 2011), and we received an affidavit of approval for the animal use protocol from Taipei Medical University (LAC-2018-0383). Male ICR mice (6 weeks) were anesthetized using a mixture containing 75% air and 3% isoflurane maintained in 25% oxygen; their external jugular veins were then cannulated with a PE-10 tube for administering the dye and drugs intravenously [28]. Venules (30–40 µm) were irradiated at a wavelength of <520 nm to produce a microthrombus. Two PTE doses (1 and 2 mg/kg) were administered 1 min following sodium fluorescein (15 µg/kg) administration, and the time required for the thrombus to occlude the microvessel (occlusion time) was recorded.

### 4.9. Statistical Analysis

Continuous variables in the experimental results are presented as the mean ± standard deviation or median (Q1–Q3) depending on whether the data are normally distributed. Values of *n* refer to the number of experiments; each experiment was conducted using different blood donors. Unpaired Student’s *t*-test or analysis of variance (ANOVA) was used to determine significant differences among the groups if the data were normally distributed. Mann–Whitney U tests and Kruskal–Wallis tests were conducted for non-normal data. When this analysis indicated significant differences, the groups were compared using the Student–Newman–Keuls method. Statistical significance was set at *p* < 0.05. 

## 5. Conclusions

This study demonstrated that PTE exhibits a strong activity against platelet activation through the inhibition of integrin α_IIb_β_3_-mediated inside-out and outside-in signaling, suggesting the potential therapeutic and prophylactic applications of PTE in thromboembolic disorders.

## Data Availability

All data generated or analyzed during this study are included in this published article.
